# Characterizing the Phenolic Compounds in Iron Walnut Oil (*Juglans sigillata* Dode) Across Chinese Regions

**DOI:** 10.3390/foods14050899

**Published:** 2025-03-06

**Authors:** Pan Gao, Kairui Chang, Shu Wang, Yuling Zheng, Jiaojiao Yin, Xinghe Zhang, Martin J. T. Reaney

**Affiliations:** 1 Key Laboratory of Edible Oil Quality and Safety, State Administration for Market Regulation, Key Laboratory for Deep Processing of Major Grain and Oil of Ministry of Education in China, College of Food Science and Engineering, Wuhan Polytechnic University, Wuhan 430023, China; 2 Department of Food Science, University of Saskatchewan, Saskatoon, SK S7N 5A2, Canada; 3 Wuhan Institute for Food and Cosmetic Control, Wuhan 430012, China

**Keywords:** iron walnut oil, UPLC-QTOF-MS, antioxidant activity, phenolic compounds, fatty acid profile, geographic variability

## Abstract

This study examines the chemical composition and antioxidant properties of iron walnut oil (IWO) from different Chinese regions, using ultra-high-performance liquid chromatography–quadrupole time-of-flight mass spectrometry for the analysis of phenolic compounds. Regional variations were identified in fatty acid profiles, with elevated α-linolenic acid levels observed in samples from cooler climates (e.g., Liaoning, sample 1) that were 60% higher than in samples from warmer regions (e.g., Sichuan, sample 2). Antioxidant properties, quantified using 1,1-diphenylpicryl phenyl hydrazine (DPPH), 2,2-azinobis-3-ethylbenzothiazoline-6-sulfonate (ABTS), and Ferric ion reducing antioxidant power (FRAP) assays, corresponded to both oil polyphenol content (up to 62.91 mg/kg) and γ-tocopherol concentrations (268.68–525.05 mg/kg). Nineteen phenolic acids and flavonoids were identified, including ellagic acid, gallic acid, p-hydroxybenzoic acid, syringic acid, vanillic acid, quercetin, caffeic acid, ferulic acid, p-coumaric acid, coniferol, and pinoresinol. This comprehensive analysis underscores the nutritional and therapeutic potential of IWO, and delineates the impact of geographic and environmental factors on its quality, providing a scientific foundation for further research and development aimed at enhancing food industry standards and exploring natural product chemistry.

## 1. Introduction

In recent years, supported by significant investments from China, the annual production of the walnut industry has seen a consistent rise, underscoring the economic and nutritional importance of this sector. In 2022, China’s walnut production exceeded 1.4 million tons, more than half of the total global production (FAOSTAT). Iron walnut (*Juglans sigillata* Dode), uniquely native to China, is predominantly cultivated in provinces such as Yunnan, Sichuan, and Gansu, as well as northeastern regions. Representing approximately 25% of China’s total walnut output, the iron walnut is distinguished by its hard shell and rich oil content, which is not only a significant contributor to regional agriculture but also of increasing research interest due to its unique nutritional and medicinal properties. Generally, iron walnut trees grow naturally without artificial intervention. Because the shell of the iron walnut is hard, the inner shell and endocarp are extremely developed; thus, the kernel yield is low, as it is difficult to separate the kernel. Therefore, it cannot readily be used as a nut and is normally processed into iron walnut oil (IWO). Geographical and environmental factors may have a direct impact on IWO quality. Climate and soil type significantly impact the chemical composition of IWO, especially with respect to the content of fatty acids and secondary metabolites [1]. It has been reported that the origin has a significant impact on walnut oil quality [2], but the quality differences in IWO from different regions have not been reported, so it is necessary to explore the impact of differences in origin on IWO quality.

IWO is rich in nutritional value. Unsaturated fatty acids constitute about 90% of its total fatty acids. Beyond lipids, walnuts are rich in essential amino acids (e.g., arginine), vitamins (e.g., folate), and minerals, contributing to cardiovascular and cognitive health [3]. It is also rich in squalene, tocopherols, polyphenol, sterols, and other minor concomitants. Consuming walnuts and walnut oil as part of a mediterranean diet can reduce serum lipids [4], while walnut oil provided to hyperlipidemic type 2 diabetic patients can improve their blood lipids [5]. Similarly, IWO can lower cholesterol in vitro [6] and improve spatial memory in rats [7]. Despite its economic and health potential, comprehensive studies on the chemical properties of and regional quality variations in IWO are limited, highlighting a significant gap in current research. Phenolic compounds, which are natural compounds with health-promoting functions [8], play a crucial role in plants. They can reduce excess reactive oxygen species produced under abiotic stress and balance oxidation homeostasis in plants by directly scavenging free radicals or regulating the expressions of related genes and enzyme activities [9]. Previous studies have shown that the content of phenolic compounds in IWO has a great impact on its quality [10], but, at present, studies on the identification and analysis of phenolic compounds in iron walnut are mainly concerned with husks, leaves, pericarps, and kernel [11,12]; there are no reports on the identification of phenolic compounds in IWO.

The existing research on walnut oil mainly focuses on the determination of total polyphenols using the Folin–Ciocalteu method, while the structure identification of phenolic compounds requires additional analysis. Previous studies have used high-performance liquid chromatography to detect eight monomer phenols in iron walnuts. These include rutin, syringic acid, chlorogenic acid, catechin, gallic acid, syringaldehyde, caffeic acid, ferulic acid, juglone, and p-coumaric acid [13]. Using liquid chromatography combined with electrospray ionization mixed linear trap quadrupole mass spectrometry (LC-LTQ Orbitrap), 120 phenolic compounds were preliminarily identified in walnut, including hydrolyzable and condensed tannins, flavonoids, and phenolic acids [14]. Sixteen polyphenol compounds, six hydroxybenzoic acid derivatives, five hydroxycinnamic acid derivatives, and five flavonoids have been identified in walnut kernels [15]. As the content of polyphenols in walnut oil is far lower than that of whole walnut, more precise detection methods are needed for qualitative analysis. The versatility and precision of UPLC-QTOF-MS in identifying polyphenolic compounds and metabolites in complex biological samples offers valuable insights for research in pharmacology, nutrition, and natural product chemistry [16]. This approach has been used to detect and identify phenolic compounds in cold-pressed camellia seed meal [17], lotus seed epicarp [18], and pomegranate peel [19].

Therefore, to systematically investigate the quality characteristics of Chinese IWO, samples from different production areas were collected to analyze IWO quality. Using ultra-high-performance liquid chromatography–quadrupole time-of-flight mass spectrometry (UPLC-QTOF-MS) and other instrumental analysis methods, the main minor compounds affecting antioxidant properties in IWO were identified, in order to clarify the composition and structure of polyphenols in IWO. By employing advanced analytical techniques, we sought to elucidate the impact of phenolics on IWO quality, thereby providing a foundation for enhancing its nutritional value.

## 2. Materials and Methods

### 2.1. Samples and Reagents

Twelve distinct IWO samples were collected from key production areas across China. All iron walnuts were harvested and processed during September and October, ensuring seasonal consistency. Sample 1 originated from Liaoning Province; samples 2 and 3 originated from the Sichuan and Chongqing regions; sample 4 originated from Shaanxi Province; samples 5 and 6 originated from Shandong Province; samples 7 and 8 originated from Gansu Province; and samples 9 to 12 originated from Yunnan Province. These samples encompass the principal IWO production areas in China, representing the diversity of the region. Iron walnuts were harvested manually, shelled, and air-dried at 25 °C for 48 h to reduce moisture content. Kernels were cold-pressed using an electric screw press (J58-630 T, Qingdao, Shandong, China) to obtain crude oil. Samples were transported in vacuum-sealed, light-protected containers within 72 h of extraction and stored at −80 °C to prevent degradation of bioactive compounds. Transportation time did not exceed 48 h for any sample.

The standards, 37-fatty acid methyl esters, tocopherols (purity > 97%), and reagents bis(trimethylsilyl)trifluoroacetamide (BSTFA) + trimethylchlorosilane (TMCS), 5α-cholestane, 2,4,6-tris(2-pyridyl) triazine (TPTZ), 2,2-diazo-bis-(3-ethylbenzothiazole-6-sulfonic acid), Trolox, 1,1-diphenylpicryl phenyl hydrazine (DPPH), and 2,2-azinobis-3-ethylbenzothiazoline-6-sulfonate (ABTS) were acquired from Sigma-Aldrich Co., Ltd. (Shanghai, China). Other necessary reagents were obtained from Sinopharm Chemical Reagent Co., Ltd. (Wuhan, Hubei, China).

### 2.2. Physicochemical Properties

The acid value (AV) and peroxide value (PV) were determined according to the methods Cd 3d-63 and Cd 8b-90, as officially recommended by AOCS. AV (mg KOH/g oil) and PV (meq O_2_/kg oil) were measured using titration, reflecting hydrolytic and oxidative degradation, respectively. The tocopherol content was analyzed according to ISO 9936. The oxidation stability index (OSI) was measured using the Metrohm Rancimat model 743 (Herisau, Switzerland), according to the AOCS Cd 12b-92 method.

### 2.3. Fatty Acid Compositon

The fatty acid composition was found using an Agilent 7890A (Palo Alto, CA, USA) gas chromatography (GC) system, as described in our previous research [20]. Each IWO sample (0.20 mg) was dissolved in 2 mL n-hexane and mixed with 0.5 mL of 2 mol/L methanolic potassium hydroxide to prepare the fatty acid methyl esters. The analytes were separated on a Trace TR-FAME capillary column (60 m × 0.25 mm, 0.25 μm, Agilent, USA). Nitrogen served as the carrier gas at a flow rate of 1 mL/min, with a split ratio of 100:1; the flame ionization detector (Agilent) and the injection temperature were set at 250 °C; and the injection volume was 1.0 μL. The GC programmed temperature gradient started at 60 °C for 3 min, rising to 170 °C at 5 °C/min. Subsequently, the temperature was maintained for 15 min, then increased to 220 °C at 2 °C/min, and then held for 10 min. The samples were identified by comparing their retention times with those of a mixture of 37-fatty acid methyl ester standards, expressing the fatty acid as the relative percentages.

### 2.4. Sterols and Squalene

The oil (0.20 g) sample was weighed and 3 mL of 2 mol/L potassium hydroxide–ethanol solution was added for saponification. The saponified solution was extracted by n-hexane until the effluent was neutral. The unsaponifiable matters were silylated with 200 µL of BSTFA + TMCS for analysis. Analyses were performed using an ISQ (Thermo Fisher, Waltham, MA, USA) mass spectrometer and a TRACE GC ULTRA gas chromatograph (Thermo Fisher) with a DB-5 capillary column (30 m × 0.25 mm, 0.25 µm, Agilent, USA) by [20]. The conditions were as follows: a helium flow rate of 1.2 mL/min, a separation ratio of 100:1, an initial column temperature and detector temperature of 280 °C, an injector temperature of 200 °C, and an injection volume of 1.0 µL. The mass of sterols and squalene in the sample was based on the peak area ratio of 5α-cholestane, and the correction factors for squalene and sterols were calculated, reporting the results in mg/kg.

### 2.5. Polyphenol Content

IWO (1.0 g) was dissolved in 5 mL n-hexane and eluted through a solid-phase extraction column (Sepax, DE, USA) with 15 mL of n-hexane. A solution of n-hexane–ethyl acetate mixture (9/1, *v*/*v*; 4 mL) was added, and the SPE column was eluted with methanol. The eluent was collected as the phenolic compounds.

Polyphenols were analyzed using the Folin–Ciocalteu method. The phenolic compounds were mixed with 0.5 mL of Folin–Ciocalteu reagent and 1 mL of Na_2_CO_3_ (10%), and incubated in the dark for 2 h. The absorbance was measured at 765 nm, and the quantitative results were calculated as mg of gallic acid equivalents per kg of sample (mg/kg).

### 2.6. Free Radical Scavenging Capacity

About 1.0 g of IWO was put into a centrifuge tube, and 5 mL of methanol was added. The tube was shaken with a DG-2500R multi-vortex mixer (Bajiu, Shanghai, China) at 2000 rpm/min in the dark for 20 min. The product was stratified with an upper methanol layer and a lower IWO layer; the upper layer was collected and used as a polar extract. The free radical scavenging capacity of the polar extract was assessed following our modified previous research method [2]. For the DPPH assay, 2 mL of the polar extract was mixed with 2 mL of DPPH–methanol. The mixture was allowed to react for 1 h in the dark at 20 °C, after which the absorbance at 517 nm was determined. For the ABTS assay, 25 mL of the ABTS solution (7 mmol/L ABTS) was mixed with 440 μL 140 mmol/L potassium persulfate and allowed to stand for 12 to 16 h in the dark, after which the absorbance at 743 nm was determined for the polar extract. For the FRAP assay, the polar extract was combined with FRAP solution, and the volume was adjusted with deionized water. The mixture was incubated at 37 °C for 10 min in the dark, centrifuged to remove solids, and the maximum absorbance was measured at 593 nm. We expressed the antioxidant capacity in terms of μmol TE/kg relative to Trolox.

### 2.7. Phenolic Compounds

Phenolic extracts were filtered through a 0.22 μm membrane and diluted 1:10 with methanol prior to injection. The phenolic compounds were analyzed using an ACQUITY UPLC PDA system (Waters, MA, USA) equipped with a BEH C18 column (2.1 mm × 50 mm, 1.7 μm, Waters) and quantified using triple quadrupole TOF mass spectrometry with electrospray ionization. The method was provided by Tang et al. [21] with some modifications. The mobile phase consisted of acetonitrile (A) and 0.1% (*v*/*v*) formic acid (B) following this elution gradient: 0–15 min, 98–80% B; 15–20 min, 80–60% B; 20–25 min, 60–20% B; 25–27 min, 20–0% B; 27.1–30 min, 0–98% B. The column temperature was 45 °C, and the flow rate was 0.3 mL/min. The ESI interface was operated in positive ion mode (ESI+) with the following settings: a collision energy of 6 eV, a capillary voltage of 3.0 kV, and a cone voltage of 20 V. The cone and desolvation gas flows were 50 and 700 L/h, respectively, with source block and desolvation temperatures at 100 °C and 400 °C. Data acquisition was conducted using Waters Mass Lynx 4.1.

### 2.8. Statistical Analysis

Statistical analyses were performed to ensure the robustness and reproducibility of our findings. Each measurement was taken in triplicate, and results are presented as mean ± standard deviation. To assess the significance of the differences among the mean values of different samples, a one-way Analysis of Variance (ANOVA) was employed, followed by Duncan’s multiple range test for post hoc analysis, allowing us to identify which specific groups differed from each other. The choice of ANOVA was predicated on its ability to handle multiple groups and to provide a clear indication of whether any of the group means are statistically significantly different from each other, assuming normal distribution of the data. Prior to conducting the ANOVA, we ensured that the data met the assumptions of normality and homogeneity of variances using Shapiro–Wilk and Levene’s tests, respectively. Multivariate linear regression (MLR) was used to analyze the obtained data. All statistical analyses were conducted using SPSS version 19.0 (IBM, IBM Corp., Armonk, NY, USA). A *p*-value of less than 0.05 was considered statistically significant, indicating strong evidence against the null hypothesis of no effect or no difference.

## 3. Results and Discussion

### 3.1. Physicochemical Properties

Table 1 presents the fundamental physicochemical parameters for IWO samples, encompassing the AV and PV. The AV for IWO varied between 0.39 and 0.74 mg/g, while the PV ranged from 0.06 to 0.24 g/100 g. Notwithstanding the notable disparities among samples sourced from distinct regions, all recorded values were beneath the prescribed limits for walnut oil in the Chinese standard, which stipulates a maximum AV of 1.0 mg/g and a PV threshold of 0.25 g/100 g. This demonstrates that the IWO samples, irrespective of their geographical origins, adhere to the established national quality benchmarks for walnut oil.

### 3.2. Fatty Acid

Table 1 elucidates the fatty acid profile of IWO, which is pivotal for appraising its economic and nutritional value. The predominant fatty acids were palmitic acid (C16:0, 2.92–9.89%), oleic acid (C18:1, 15.80–22.39%), linolenic acid (C18:2, 51.97–68.81%), and α-linoleic acid (C18:3n3, 4.16–17.23%). The IWO samples exhibited a high concentration of polyunsaturated fatty acids (PUFA, 65.21–79.68%), which are acknowledged for their salutary effects on human health. Interestingly, the analysis also revealed the presence of several minor fatty acids in IWO, including lauric acid (C12:0), tridecanoic acid (C13:0), myristic acid (C14:0), pentadecanoic acid (C15:0), palmitoleic acid(C16:1), heptadecanoic acid (C17:0), heptadecenoic acid (C17:1), and arachidic acid (C20:0). Notably, the fatty acids of C13:0, C15:0, C17:0, and C17:1 were identified in IWO for the first time, highlighting their unique properties and potential health benefits that may augment the nutritional profile of IWO.

A comparative analysis of fatty acid compositions in IWO sourced from various regions identified significant regional discrepancies impacting the fatty acid profiles and concentrations. Notably, the proportions of C16:0 and C18:1 fatty acid in sample 1 were substantially lower, recorded at 2.92% and 15.80%, respectively, compared to the counterparts in other samples. Conversely, sample 1 exhibited a pronounced increase in C18:2 fatty acid content, marking a 16.84% elevation over that in sample 8. This variation could be attributed to the doubled content of C18:3n3 in sample 8 compared to others, which, despite the disparity, led to a comparable total polyunsaturated fatty acid (PUFA) content across the samples.

The pronounced regional variations observed in the fatty acid profiles of IWO underscore the influence of environmental factors such as climate and soil type. For instance, oils from colder regions like northeastern China (e.g., sample 1 from Liaoning Province) showed a higher content of unsaturated fatty acids, which might be a natural adaptation to enhance fluidity at lower temperatures. Conversely, oils from warmer southern regions (e.g., sample 2 from Sichuan Province) exhibited increased saturated fatty acids, potentially reflecting a metabolic response to environmental stressors such as higher temperatures and more intense sunlight exposure. Our results demonstrate clear geographic influences on the biochemical composition of IWO, suggesting that tailored agricultural practices could significantly enhance oil quality. By comparing our findings with those from studies in similar climates [22], it becomes evident that regional adaptation strategies for walnut cultivation could yield substantial improvements in both the antioxidant and fatty acid profiles of IWO. Future research should explore the mechanistic basis of these regional differences, potentially guiding the development of targeted cultivation techniques.

### 3.3. Minor Compounds

Additionally, the minor compound analysis in IWO (Table 1) reveals a spectrum of tocopherols, encompassing α-, β-, γ-, and δ-tocopherols, with total content spanning 268.68 to 525.05 mg/kg. Predominantly, γ-tocopherol constitutes 84–99% of the total tocopherol concentration. Sample 2 has the highest levels of α-tocopherol (78.58 mg/kg) and γ-tocopherol (440.27 mg/kg), contributing to its superior total tocopherol content, which is a third higher than that of other samples. The absence of α-tocopherol in samples 1, 3, and 10, despite previous detections in IWO, could be ascribed to genetic and environmental variations [23]. The polyphenol content of IWO produced in different regions varies significantly (1.22–62.91 mg/kg), with sample 5 having the highest polyphenol content (62.91 mg/kg), followed by sample 6 (55.15 mg/kg), while samples 1, 2, 4, 10, and 12 have polyphenol contents below 10 mg/kg.

The sterol profile in IWO predominantly includes β-Sitosterol (1153.29–3737.97 mg/kg), Δ5- avenasterol (101.19–418.24 mg/kg), campesterol (61.35–1135.52 mg/kg), and stigmasterenol (71.91–203.58 mg/kg), with campestanol and ergost-5-en-3β-ol exclusively identified in sample 2, marking a first in IWO detections. This unique sterol composition in sample 2 could be attributed to Sichuan’s distinct topography and geography, enriching its IWO with diverse lipid components.

Squalene, a natural lipid with an isoprene structure and part of the terpenoid class, is known for its anti-inflammatory, antioxidative, and antiatherosclerotic properties [24] and exhibits significant variability across different production regions. The squalene content in IWO, ranging from 2.03 to 27.16 mg/kg, underscores notable regional differences, with samples 2 (27.16 mg/kg) and 5 (17.87 mg/kg) showing significantly elevated levels compared to prior reports [20]. This variability underscores the influence of regional environmental conditions on the biosynthesis pathways of lipidic compounds in IWO. 

### 3.4. Antioxidant Ability

The results of IWO’s antioxidant ability are shown in Table 1. The OSI and radical scavenging capacities of IWO from different origins varied greatly, with sample 5 showing the strongest OSI, DPPH, ABTS, and FRAP radical scavenging capacities of 6.45 h, 519.65 μmol TE/100 g, 683.26 μmol TE/100 g, and 158.70 μmol TE/100 g, respectively. Although the principles of the four detection methods are different, OSI tracks the oxidation degree by monitoring the formation of specific oxidation products and secondary products in the sample, mainly detecting increases in conductivity or polar substances in the sample. DPPH radical characterizes the ability of antioxidants to provide hydrogen atoms. ABTS expresses its antioxidant ability by reducing with hydrogen peroxide or other oxidants, while FRAP evaluates its ability to reduce Fe^3+^ to Fe^2+^. In general, the antioxidant capacity of samples 2, 6, 9, and 11 was inferior to that of sample 5, showing relatively good antioxidant performance, while sample 1 was the weakest.

### 3.5. Multivariate Linear Regression and Relative Correlation Analysis

The MLR analysis determines the relationship between major fatty acids, minor components, and their impact on antioxidant activities; the details of these correlations can be seen in Table 2. This statistical approach is pivotal for unraveling how these compounds act individually and together, against oxidative stress. The results from this study demonstrate polyphenol antioxidant potency. In the DPPH assay, the model explains a remarkable 84.4% of the variance in antioxidant capacity, underscoring the substantial role of polyphenol in combating free radicals. This is further corroborated by the statistical significance of polyphenol as a predictor in all assays, with *p*-values indicating a less than 0.01% chance that these findings were due to random variation. The statistical evidence indicates the role polyphenols play in antioxidant defenses. Similarly, γ-tocopherol shows significant contributions to antioxidant activity in specific contexts, notably in the DPPH and OSI assays. These findings suggest that, while polyphenols have a more universally potent antioxidant effect, γ-tocopherol’s impact is significant but varies depending on the assay, pointing to its nuanced role in the antioxidant network.

**Figure 1:**
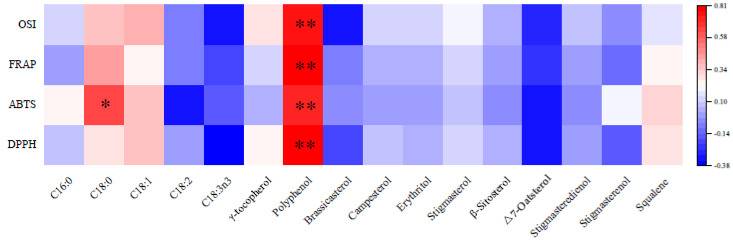
The relative correlation analysis of iron walnut oil. * *p* < 0.05, ** *p* < 0.01.

Figure 1 elucidates the intricate relationships between various compounds and their impact on antioxidant activities in OSI, FRAP, ABTS, and DPPH assays. The results indicate a strong positive correlation between polyphenols and all measured antioxidant activities. This suggests that polyphenols could significantly mitigate oxidation. In contrast to polyphenols, γ-tocopherol might not show a significant correlation with antioxidant activities. This absence of a strong relationship suggests that while γ-tocopherol has recognized antioxidant properties, its impact might be less pronounced or more context-dependent than polyphenols. The fatty acids and sterols might show a weak correlation with antioxidant activities.

### 3.6. Polyphenol Compounds

**Figure 2:**
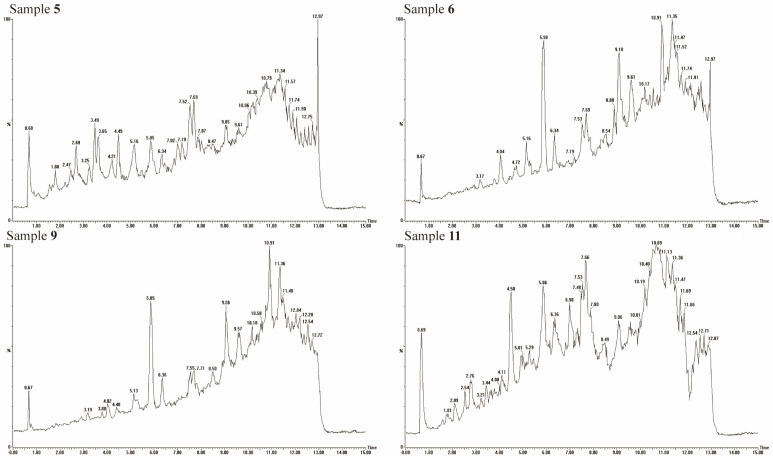
Total ion flow chart of phenolic compounds in iron walnut oil.

To elucidate the phenolic composition of IWO, UPLC-QTOF-MS analysis was conducted on samples 5, 6, 9, and 11, which exhibited the highest polyphenol content. The main polyphenols in IWO were identified by database screening and comparison of retention times, accurate mass, and fragment ions by authentic standards. The total ion chromatograms, depicted in Figure 2, revealed distinct variations among the four spectra, indicating diverse polyphenolic profiles across the IWO samples. Preliminary spectral analysis, supplemented by UV and MS data, identified nineteen phenolic entities, encompassing twelve phenolic acids, two lignans, two flavonoids, and three sesquiterpenes, as summarized in Table 3. Notably, sample 5 was distinguished by both the highest polyphenolic concentration and diversity.

Compound **1** was discerned by its deprotonated molecular ion [M-H]^−^ at *m*/*z* 341, with fragment ions at *m*/*z* 179 indicative of hexoside moiety loss, representing monosaccharide fragments [25]. The presence of hexose was corroborated by typical hexose fragments at *m*/*z* 71, 89, 113, 119, and 131 [26]. Phenolic acids, categorized into hydroxybenzoic and hydroxycinnamic acids, exhibit distinct fragmentation patterns. Hydroxybenzoic acids typically lose a CO_2_ unit, yielding [M-H-44]^−^ ions, while hydroxycinnamic acids produce characteristic ions upon pyrolysis. Compounds **2**, **3**, **8**, and **12**, corresponding to hydroxybenzoic acids like gallic, p-hydroxybenzoic, syringic, and vanillic acids, displayed parent ions at *m*/*z* 137 and *m*/*z* 167, with CO_2_ loss fragments at *m*/*z* 93 and *m*/*z* 123, respectively [27]. Hydroxycinnamic acid representatives, specifically compounds **4**, **5**, and **13** (p-coumaric, ferulic, and caffeic acids), mirrored hydroxybenzoic acid fragmentation, generating ions at *m*/*z* 117 and 135 from parent ions *m*/*z* 163 and 179. Compound **5** also released a methyl group and CO_2_, yielding fragments *m*/*z* 178 [M-H-CH_3_]^−^ and *m*/*z* [28].

**Figure 3:**
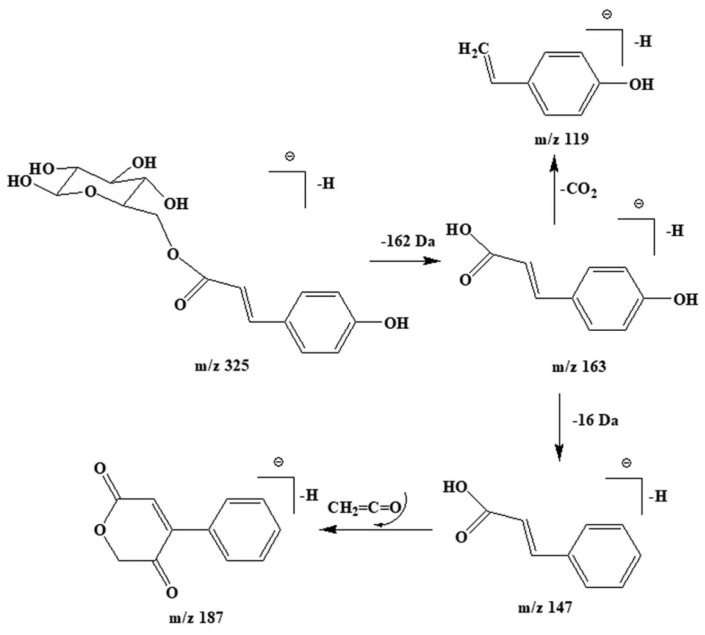
The proposed cleavage pathways for compound **19**.

Caffeic acid’s signature fragments (*m*/*z* 179 and 135) and quinic acid’s parent ion (*m*/*z* 179) led to the identification of compound **16** as chlorogenic acid, a caffeic and quinic acid condensate, with [M-H]^−^ at *m*/*z* 353. Compound **19**, with *m*/*z* 325, generated the notable fragments *m*/*z* 163 [M-H-162]^−^ and *m*/*z* 119, suggesting p-coumaric acid and hexose units, identified as p-coumaric hexose, possibly through p-coumaric acylation with hexose. The possible cleavage pathways of this compound are shown in Figure 3. IWO’s lignans, predominantly coniferol and pinoresinol (compounds **6** and **17**), were identified by their spectra. Compound 6 exhibited a deprotonated ion at *m*/*z* 180, with guaiacyl fragments at *m*/*z* 178, 136, 124, confirming it as coniferol [29], a lignin monomer. Compound **17**’s [M-H]^−^ at *m*/*z* 357 exhibited typical lignin cleavages, yielding primary ion *m*/*z* 151 (o-methoxyphenyl) and base peak *m*/*z* 136 upon demethylation [30].

**Figure 4:**
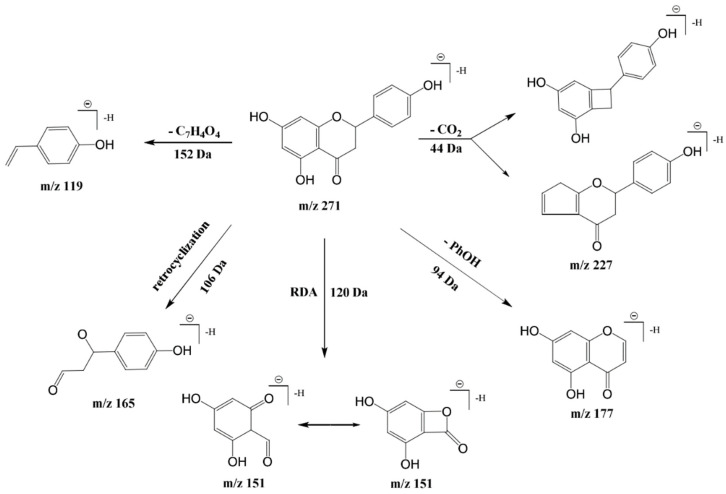
The proposed cleavage pathways for compound **14**.

Flavonoid compound **14**, characterized by two benzene rings and a pyran ring, displayed a [M-H]^−^ parent ion at *m*/*z* 271. Collision-induced fragmentation led to a CO_2_ loss yielding *m*/*z* 227 [M-H-CO_2_]^−^, further losing a phenolic hydroxyl to produce *m*/*z* 177 [M-H-PhOH]^−^. The reverse cyclization and Retro Diels-Alder cleavage of flavonoid aglycones generated *m*/*z* 165 and 151 fragments, respectively, with *m*/*z* 119 fragments aligning with naringenin’s fragmentation pattern, suggesting that compound **14’s** identity was naringenin [31]. The proposed cleavage pathways are shown in Figure 4. Compound **10** was confirmed to be abscisic acid by its [M-H]^−^ at *m*/*z* 263 [32], retention time, and MS2 fragmentation. Compound **11’s** parent ion [M-H]^−^ at *m*/*z* 425, with fragments at *m*/*z* 263, 153, and 219, and lacking a 162 Da fragment, indicated a hexosylated abscisic acid derivative, identified as abscisic acid-o-hexoside.

Quercetin and ellagic acid differentiation was achieved through MS2 fragmentation in negative ion mode, with quercetin yielding *m*/*z* 179 and 151, and ellagic acid producing *m*/*z* 257, 229, and 185 ions [33], identifying compounds **15** and **20** as ellagic acid and quercetin, respectively. Lastly, compound **18**, with a [M-H]^−^ ion at *m*/*z* 491, underwent fragmentation yielding a *m*/*z* 329 ion, indicative of caffeic acid loss (162 Da), with caffeic acid and its decarboxylate products generating low-intensity signals [34]. The MS2 fragmentation of *m*/*z* 329 revealed ions at *m*/*z* 311 (caffeoyl tartaric acid), 149 (tartaric acid), and 135 (decarboxycaffeic acid), identifying the substance as caffeoyl dihydroxyphenyl lactyl tartaric acid.

## 4. Conclusions

This study comprehensively analyzed the chemical composition and antioxidant properties of IWO from different regions in China, underscoring its nutritional and bioactive significance. Our findings reveal that IWO is rich in unsaturated fatty acids and bioactive compounds such as squalene, tocopherols, and polyphenols, which significantly contribute to human health. Our comprehensive analysis confirms that geographic and climatic conditions play a critical role in shaping the fatty acid and polyphenol profiles of IWO. This study establishes UPLC-QTOF-MS as a robust tool for profiling IWO phenolics. Regional climate-driven variations in composition underscore the potential for targeted cultivation to maximize health benefits.

Our findings highlight the significant influence of geographic and climatic factors on IWO composition. While regional agricultural practices were not explicitly examined, the observed variations in fatty acids and phenolics suggest that environmental adaptation strategies could further enhance oil quality. Future studies should directly evaluate cultivation techniques to optimize bioactive compound synthesis. In summary, this research not only enhances our understanding of the chemical properties of IWO but also supports its application in the health food sector, hoping to inspire further comprehensive studies on iron walnut and its products to fully realize its potential in the global health food market.

## Figures and Tables

**Table 1:** The physiochemical properties, fatty acid composition, minor compounds content, and antioxidant ability of iron walnut oil.

	1	2	3	4	5	6	7	8	9	10	11	12
AV (mg/g)	0.39 ± 0.01 ^h^	0.42 ± 0.00 ^g^	0.41 ± 0.00 ^g^	0.46 ± 0.00 ^f^	0.54 ± 0.01 ^d^	0.54 ± 0.00 ^d^	0.62 ± 0.01 ^c^	0.62 ± 0.01 ^c^	0.48 ± 0.00 ^e^	0.63 ± 0.01 ^c^	0.74 ± 0.03 ^a^	0.68 ± 0.00 ^b^
PV (g/100 g)	0.06 ± 0.00 ^g^	0.07 ± 0.00 ^f^	0.06 ± 0.00 ^g^	0.09 ± 0.00 ^cd^	0.09 ± 0.00 ^c^	0.08 ± 0.00 ^de^	0.09 ± 0.00 ^cd^	0.08 ± 0.00 ^de^	0.07 ± 0.00 ^ef^	0.07 ± 0.00 ^f^	0.24 ± 0.00 ^a^	0.23 ± 0.00 ^b^
Fatty acid (%)												
C12:0	0.05 ± 0.01 ^f^	0.08 ± 0.01 ^e^	0.05 ± 0.00 ^f^	0.14 ± 0.01 ^b^	0.05 ± 0.00 ^f^	0.05 ± 0.00 ^f^	0.12 ± 0.01 ^c^	0.10 ± 0.01 ^d^	0.05 ± 0.00 ^f^	0.16 ± 0.01 ^a^	0.05 ± 0.00 ^f^	0.05 ± 0.00 ^f^
C13:0	0.11 ± 0.00 ^a^	0.11 ± 0.01 ^a^	0.10 ± 0.00 ^ab^	0.08 ± 0.01 ^c^	0.10 ± 0.00 ^ab^	0.10 ± 0.01 ^ab^	0.10 ± 0.00 ^ab^	0.11 ± 0.01 ^ab^	0.09 ± 0.00 ^bc^	0.09 ± 0.00 ^bc^	0.10 ± 0.01 ^ab^	0.10 ± 0.01 ^ab^
C14:0	N.D. ^c^	0.03 ± 0.01 ^ab^	0.02 ± 0.00 ^b^	0.02 ± 0.00 ^b^	0.03 ± 0.01 ^ab^	0.02 ± 0.00 ^b^	0.03 ± 0.01 ^c^	0.03 ± 0.01 ^ab^	N.D. ^c^	0.03 ± 0.00 ^ab^	0.03 ± 0.01 ^ab^	N.D. ^c^
C15:0	N.D. ^c^	N.D. ^c^	0.03 ± 0.01 ^b^	N.D. ^c^	N.D. ^c^	N.D. ^c^	0.02 ± 0.00 ^b^	N.D. ^c^	N.D. ^c^	0.02 ± 0.00 ^b^	0.04 ± 0.01 ^a^	N.D. ^c^
C16:0	2.92 ± 0.03 ^i^	9.89 ± 0.15 ^a^	3.38 ± 0.19 ^h^	5.89 ± 0.02 ^f^	6.15 ± 0.12 ^e^	5.45 ± 0.02 ^g^	5.73 ± 0.08 ^f^	5.85 ± 0.02 ^f^	5.37 ± 0.01 ^g^	6.40 ± 0.01 ^d^	7.68 ± 0.02 ^c^	7.92 ± 0.09 ^b^
C16:1	0.04 ± 0.01 ^c^	0.13 ± 0.01 ^b^	0.05 ± 0.01 ^c^	0.06 ± 0.00 ^c^	0.05 ± 0.01 ^c^	0.06 ± 0.01 ^c^	0.07 ± 0.00 ^c^	0.05 ± 0.00 ^c^	0.06 ± 0.00 ^c^	0.12 ± 0.01 ^b^	0.20 ± 0.02 ^a^	0.19 ± 0.04 ^a^
C17:0	N.D. ^c^	0.05 ± 0.01 ^a^	N.D. ^c^	0.04 ± 0.00 ^ab^	0.06 ± 0.01 ^a^	0.05 ± 0.00 ^ab^	0.04 ± 0.01 ^ab^	0.05 ± 0.01 ^ab^	0.06 ± 0.01 ^a^	0.04 ± 0.01 ^ab^	0.04 ± 0.00 ^ab^	0.04 ± 0.01 ^b^
C17:1	0.03 ± 0.00 ^a^	0.03 ± 0.01 ^a^	N.D. ^b^	0.03 ± 0.01 ^a^	N.D. ^b^	0.04 ± 0.00 ^a^	0.04 ± 0.02 ^a^	0.03 ± 0.00 ^a^	0.04 ± 0.00 ^a^	0.04 ± 0.01 ^a^	0.04 ± 0.01 ^a^	N.D. ^b^
C18:0	1.07 ± 0.00 ^g^	2.25 ± 0.03 ^f^	1.09 ± 0.03 ^g^	3.00 ± 0.00 ^c^	3.72 ± 0.03 ^a^	2.49 ± 0.01 ^d^	2.97 ± 0.03 ^c^	3.11 ± 0.01 ^b^	2.49 ± 0.04 ^d^	2.48 ± 0.00 ^d^	2.44 ± 0.01 ^de^	2.40 ± 0.02 ^e^
C18:1	15.80 ± 0.03 ^g^	21.87 ± 1.02 ^ab^	17.62 ± 0.00 ^f^	19.91 ± 0.02 ^c^	18.84 ± 0.03 ^de^	22.42 ± 0.01 ^a^	22.39 ± 0.04 ^a^	21.26 ± 0.03 ^b^	22.43 ± 0.00 ^a^	20.41 ± 0.01 ^c^	19.08 ± 0.04 ^d^	18.38 ± 0.04 ^e^
C18:2	68.81 ± 0.01 ^a^	61.05 ± 1.11 ^e^	64.99 ± 0.16 ^b^	62.15 ± 0.02 ^c^	61.26 ± 0.08 ^de^	61.95 ± 0.04 ^cd^	59.14 ± 0.09 ^f^	51.97 ± 0.01 ^g^	62.00 ± 0.04 ^cd^	61.80 ± 0.01 ^cde^	61.28 ± 0.02 ^de^	61.87 ± 0.07 ^cd^
C18:3n6	0.04 ± 0.00b ^cd^	0.05 ± 0.01 ^b^	0.05 ± 0.01 ^b^	0.04 ± 0.01 ^bcd^	0.05 ± 0.01 ^bc^	N.D. ^e^	0.03 ± 0.00 ^cd^	0.07 ± 0.01 ^a^	0.04 ± 0.01 ^cd^	0.03 ± 0.00 ^d^	0.04 ± 0.00 ^cd^	N.D. ^e^
C18:3n3	10.86 ± 0.01 ^c^	4.16 ± 0.23 ^i^	12.41 ± 0.03 ^b^	8.53 ± 0.01 ^f^	9.41 ± 0.02 ^d^	7.20 ± 0.03 ^h^	9.04 ± 0.02 ^e^	17.23 ± 0.04 ^a^	7.17 ± 0.05 ^h^	8.30 ± 0.00 ^g^	8.88 ± 0.00 ^e^	8.96 ± 0.01 ^de^
C20:0	0.23 ± 0.02 ^a^	0.16 ± 0.01 ^b^	N.D. ^g^	0.06 ± 0.01 ^ef^	0.11 ± 0.00 ^c^	N.D. ^g^	0.09 ± 0.01 ^d^	0.07 ± 0.01 ^de^	0.05 ± 0.02 ^f^	0.07 ± 0.01 ^def^	N.D. ^g^	N.D. ^g^
C20:1	0.03 ± 0.01 ^g^	0.16 ± 0.00 ^cde^	0.22 ± 0.03 ^ab^	0.15 ± 0.00 ^de^	0.19 ± 0.00 ^bc^	0.18 ± 0.01 ^cd^	0.24 ± 0.00 ^a^	0.15 ± 0.01 ^de^	0.18 ± 0.01 ^cd^	0.13 ± 0.01 ^ef^	0.13 ± 0.03 ^ef^	0.10 ± 0.01 ^f^
SFA	4.38 ± 0.05 ^g^	12.57 ± 0.16 ^a^	4.66 ± 0.22 ^f^	9.23 ± 0.03 ^d^	10.21 ± 0.16 ^c^	8.16 ± 0.04 ^e^	9.10 ± 0.13 ^d^	9.30 ± 0.03 ^d^	8.09 ± 0.07 ^e^	9.29 ± 0.00 ^d^	10.36 ± 0.04 ^bc^	10.51 ± 0.11 ^b^
MUFA	15.90 ± 0.05 ^f^	22.19 ± 1.04 ^a^	21.41 ± 0.23 ^b^	20.15 ± 0.01 ^c^	19.08 ± 0.03 ^de^	22.69 ± 0.02 ^a^	22.74 ± 0.03 ^a^	21.49 ± 0.02 ^b^	22.71 ± 0.02 ^a^	20.69 ± 0.02 ^c^	19.44 ± 0.03 ^d^	18.76 ± 0.06 ^e^
PUFA	79.68 ± 0.00 ^a^	65.21 ± 0.88 ^g^	74.21 ± 0.61 ^b^	70.68 ± 0.03 ^cd^	70.67 ± 0.10 ^cd^	69.15 ± 0.07 ^e^	68.18 ± 0.11 ^f^	69.20 ± 0.05 ^e^	69.17 ± 0.01 ^e^	70.11 ± 0.01 ^d^	70.16 ± 0.01 ^d^	70.83 ± 0.06 ^c^
Tocopherol (mg/g)												
α-tocopherol	N.D. ^h^	78.58 ± 0.17 ^a^	N.D. ^h^	3.57 ± 0.12 ^g^	26.36 ± 0.76 ^b^	11.78 ± 0.00 ^d^	5.09 ± 0.62 ^fg^	7.96 ± 0.30 ^e^	23.74 ± 2.85 ^c^	N.D. ^h^	11.78 ± 0.97 ^d^	6.00 ± 0.66 ^ef^
β-tocopherol	N.D. ^d^	N.D. ^d^	N.D. ^d^	N.D. ^d^	2.26 ± 0.41 ^b^	4.84 ± 0.09 ^a^	N.D. ^d^	1.24 ± 0.36 ^c^	1.15 ± 0.82 ^c^	1.75 ± 0.31 ^bc^	1.65 ± 0.23 ^bc^	N.D. ^d^
γ-tocopherol	329.73 ± 2.35 ^d^	440.27 ± 4.85 ^a^	355.54 ± 1.33 ^b^	308.72 ± 2.91 ^e^	327.45 ± 0.61 ^d^	343.22 ± 2.24 ^c^	245.79 ± 3.79 ^g^	263.92 ± 2.40 ^f^	305.77 ± 3.16 ^e^	350.15 ± 2.59 ^bc^	304.22 ± 5.50 ^e^	333.95 ± 4.29 ^d^
δ-tocopherol	3.54 ± 0.79 ^e^	6.19 ± 0.92 ^d^	13.35 ± 0.93 ^c^	12.27 ± 0.50 ^c^	26.78 ± 0.65 ^a^	7.23 ± 0.37 ^d^	17.81 ± 1.41 ^b^	16.97 ± 1.10 ^b^	6.98 ± 0.61 ^d^	18.63 ± 0.64 ^b^	16.84 ± 0.34 ^b^	18.38 ± 0.71 ^b^
Total tocopherol	333.27 ± 3.14 ^ef^	525.05 ± 3.76 ^a^	368.89 ± 2.2^5 c^	324.56 ± 2.28 ^f^	382.85 ± 1.23 ^b^	367.07 ± 1.96 ^cd^	268.68 ± 5.82 ^h^	290.09 ± 3.56 ^g^	337.64 ± 5.79 ^e^	370.54 ± 3.54 ^c^	334.48 ± 6.58 ^e^	358.33 ± 4.33 ^d^
Polyphenol (mg/g)	4.10 ± 0.35 ^j^	1.22 ± 0.00 ^k^	10.01 ± 0.41 ^f^	6.45 ± 0.35 ^i^	62.91 ± 0.21 ^a^	55.15 ± 0.48 ^b^	12.10 ± 0.21 ^e^	22.24 ± 0.28 ^d^	42.98 ± 0.28 ^c^	7.62 ± 0.07 ^h^	42.76 ± 0.69 ^c^	8.51 ± 0.07 ^g^
Sterols (mg/g)												
Brassicasterol	70.83 ± 1.00 ^c^	62.11 ± 0.36 ^f^	73.42 ± 3.41 ^b^	66.21 ± 0.05 ^e^	69.29 ± 0.86 ^cd^	66.64 ± 0.13 ^de^	143.01 ± 1.05 ^a^	67.10 ± 1.15 ^de^	66.28 ± 0.13 ^e^	67.84 ± 0.53 ^de^	66.18 ± 0.49 ^e^	66.39 ± 0.28 ^e^
Campesterol	145.11 ± 2.55 ^d^	1135.52 ± 1.38 ^a^	213.53 ± 1.17 ^c^	78.40 ± 0.71 ^g^	113.01 ± 0.35 ^e^	66.12 ± 0.62 ^i^	265.72 ± 1.54 ^b^	216.61 ± 4.35 ^c^	71.93 ± 0.41 ^h^	76.27 ± 1.76 ^g^	94.48 ± 1.44 ^f^	61.35 ± 1.66 ^j^
Campestanol	N.D. ^b^	79.82 ± 2.01 ^a^	N.D. ^b^	N.D. ^b^	N.D. ^b^	N.D. ^b^	N.D. ^b^	N.D. ^b^	N.D. ^b^	N.D. ^b^	N.D. ^b^	N.D. ^b^
Stigmasterol	12.57 ± 0.28 ^e^	331.53 ± 1.42 ^a^	6.61 ± 0.11 ^h^	12.02 ± 0.60 ^ef^	34.87 ± 1.57 ^c^	10.71 ± 0.73 ^f^	26.11 ± 0.46 ^d^	54.34 ± 0.82 ^b^	7.84 ± 0.06 ^gh^	7.64 ± 0.16 ^gh^	10.60 ± 0.11 ^f^	8.55 ± 0.4 ^g^
Ergost-5-en-3β-ol	N.D. ^b^	32.81 ± 0.64 ^a^	N.D. ^b^	N.D. ^b^	N.D. ^b^	N.D. ^b^	N.D. ^b^	N.D. ^b^	N.D. ^b^	N.D. ^b^	N.D. ^b^	N.D. ^b^
Erythritol	20.53 ± 0.59 ^b^	85.28 ± 2.59 ^a^	17.18 ± 0.36 ^cd^	18.77 ± 0.54 ^bc^	17.85 ± 1.06 ^cd^	10.49 ± 0.43 ^f^	18.10 ± 0.81 ^cd^	18.20 ± 0.15 ^cd^	9.77 ± 0.07 ^f^	14.52 ± 0.36 ^e^	16.27 ± 0.36 ^de^	9.90 ± 0.68 ^f^
β-Sitosterol	1730.98 ± 2.65 ^c^	3737.97 ± 6.26 ^a^	1960.08 ± 3.60 ^b^	1358.05 ± 3.53 ^h^	1393.35 ± 4.28 ^g^	1170.97 ± 3.32 ^j^	1690.97 ± 2.21 ^d^	1452.22 ± 2.14 ^f^	1166.11 ± 3.44 ^j^	1275.03 ± 1.45 ^i^	1602.59 ± 4.32 ^e^	1153.29 ± 4.58 ^k^
△5-Avenasterol	348.29 ± 0.73 ^b^	418.24 ± 1.84 ^a^	327.42 ± 1.14 ^c^	234.46 ± 1.22 ^e^	199.16 ± 0.53 ^f^	112.18 ± 1.62 ^j^	181.62 ± 2.31 ^h^	313.59 ± 0.43 ^d^	101.19 ± 1.74 ^k^	144.94 ± 1.98 ^i^	187.19 ± 0.49 ^g^	187.86 ± 1.73 ^g^
Stigmasteredienol	32.56 ± 0.28 ^c^	115.03 ± 0.70 ^a^	27.91 ± 0.39 ^d^	23.74 ± 0.02 ^e^	21.68 ± 0.20 ^f^	13.89 ± 1.13 ^h^	23.37 ± 0.13 ^e^	36.30 ± 0.44 ^b^	14.05 ± 0.97 ^h^	13.67 ± 0.92 ^h^	17.60 ± 0.56 ^g^	16.58 ± 0.32 ^g^
Stigmasterenol	71.91 ± 0.28 ^i^	203.58 ± 1.46 ^b^	168.36 ± 0.67 ^d^	191.01 ± 1.37 ^c^	157.16 ± 2.15 ^e^	117.38 ± 1.90 ^h^	125.09 ± 2.45 ^g^	275.90 ± 1.10 ^a^	141.71 ± 1.45 ^f^	142.12 ± 0.16 ^f^	191.41 ± 0.18 ^c^	192.18 ± 0.50 ^c^
△7-Oatsterol	N.D. ^c^	54.55 ± 2.23 ^a^	N.D. ^c^	N.D. ^c^	N.D. ^c^	N.D. ^c^	N.D. ^c^	16.45 ± 0.12 ^b^	N.D. ^c^	N.D. ^c^	N.D. ^c^	N.D. ^c^
Squalene (mg/g)	6.87 ± 0.56 ^f^	9.65 ± 0.26 ^d^	27.16 ± 1.12 ^a^	2.03 ± 0.10 ^g^	17.87 ± 0.36 ^b^	6.10 ± 0.10 ^f^	2.42 ± 0.03 ^g^	6.07 ± 0.09 ^f^	6.02 ± 0.16 ^f^	8.45 ± 0.48 ^e^	14.10 ± 0.14 ^c^	14.33 ± 0.32 ^c^
OSI (h)	1.77 ± 0.04 ^k^	4.56 ± 0.01 ^d^	3.45 ± 0.07 ^e^	2.87 ± 0.01 ^g^	6.45 ± 0.06 ^a^	6.11 ± 0.00 ^b^	2.14 ± 0.06 ^j^	3.05 ± 0.06 ^f^	5.38 ± 0.07 ^c^	2.33 ± 0.00 ^i^	2.84 ± 0.04 ^g^	2.58 ± 0.06 ^h^
Free radical scavenging capacity (μmol TE/100 g)												
DPPH	360.24 ± 3.57 ^g^	442.95 ± 4.28 ^c^	429.02 ± 3.57 ^d^	370.62 ± 9.27 ^fg^	519.65 ± 7.13 ^a^	493.64 ± 6.42 ^b^	389.64 ± 2.14 ^e^	377.59 ± 1.43 ^f^	488.13 ± 2.14 ^b^	390.06 ± 3.57 ^e^	423.94 ± 5.71 ^d^	367.82 ± 3.57 f^g^
ABTS	213.58 ± 1.32 ^i^	440.84 ± 6.59 ^c^	431.08 ± 1.32 ^d^	390.44 ± 1.32 ^g^	683.26 ± 1.32 ^a^	475.09 ± 2.64 ^b^	410.53 ± 5.28 ^ef^	408.69 ± 1.32 ^f^	468.41 ± 7.92 ^b^	402.48 ± 2.64 ^f^	419.23 ± 7.91 ^e^	368.88 ± 2.64 ^h^
FRAP	70.27 ± 0.00 ^gh^	98.40 ± 3.26 ^d^	90.48 ± 1.63 ^e^	73.58 ± 1.63 ^g^	158.70 ± 2.85 ^a^	118.86 ± 0.41 ^b^	87.31 ± 1.22 ^e^	81.73 ± 3.26 ^f^	113.04 ± 2.04 ^c^	66.72 ± 0.81 ^h^	78.55 ± 1.22 ^f^	71.90 ± 3.26 ^g^

All data represent the mean of three replications (Mean ± SD). The superscript letters indicate the statistical difference in rows in significant level at 5%. N.D. means not detect. Samples 1–12 correspond to Liaoning (1), Sichuan (2), Chongqing (3), Shaanxi (4), Shandong (5–6), Gansu (7–8), and Yunnan (9–12). SFA: Saturated fatty acid, MUFA: Monounsaturated fatty acid, PUFA: Polyunsaturated fatty acid.

**Table 2:** Multiple linear regression analysis between antioxidant ability and IWO components.

Dependent Variable	R^2^	Adjusted R^2^	Variable	R	Standard Error	t	Significance (Two-Tails *p*)	Equation
DPPH	0.844	0.810	(constant)	3.113 × 10^−16^	0.126	0.000	1.000	Y = 3.113 × 10^−16^ + 0.904 (Polyphenol) + 0.439 (γ-Tocopherol)
			Polyphenol	0.904	0.134	6.724	0.000	
			γ-Tocopherol	0.439	0.134	3.265	0.010	
ABTS	0.516	0.467	(constant)	−1.585 × 10^−16^	0.211	0.000	1.000	Y = −1.585 × 10^−16^ + 0.718 (Polyphenol)
			Polyphenol	0.718	0.220	3.264	0.009	
FRAP	0.612	0.573	(constant)	2.788 × 10^−16^	0.189	0.000	1.000	Y = 2.788 × 10^−16^ + 0.782 (Polyphenol)
			Polyphenol	0.782	0.197	3.973	0.003	
OSI	0.796	0.750	(constant)	−3.083 × 10^−16^	0.144	0.000	1.000	Y = −3.083 × 10^−16^ + 0.862 (Polyphenol) + 0.471 (γ-Tocopherol)
			Polyphenol	0.862	0.154	5.594	0.000	
			γ-Tocopherol	0.471	0.154	3.061	0.014	

**Table 3:** Preliminary identification of phenolic substances in iron walnut oil.

No.	Retention Time (min)	MW	[M-H] (*m*/*z*)	Fragment Irons	Compounds	Molecular Formula	Samples
1	0.725	342	341	179, 131, 119, 113, 89, 71	Hexose polymer		5, 6, 9, 11
2	0.932	170	169	137, 87	Gallic acid	C_7_H_6_O_5_	5, 6, 9, 11
3	1.516	138	137	93	P-hydroxybenzoic acid	C_7_H_6_O_3_	5, 6, 9, 11
4	2.129	164	163	117	P-coumaric acid	C_10_H_10_O_3_	5, 6, 9, 11
5	2.475	194	193	178, 134	Ferulic acid	C_10_H_10_O_4_	5, 6, 11
6	2.580	181	180	178, 136, 124	Pine cypress alcohol	C_10_H_12_O_3_	5, 6, 9, 11
7	2.687	182	181	137, 109	High vanillic acid	C_9_H_10_O_4_	5, 6
8	2.696	198	197	182, 167, 153	Eugenoic acid	C_9_H_10_O_5_	5, 6
9	2.908	174	173	145	Hydroquinone	C_10_H_6_O_3_	5, 6, 9, 11
10	3.246	264	263	219, 204, 201, 153	Abscisic acid	C_15_H_20_O_4_	5, 6, 9, 11
11	3.478	426	425	425, 263, 219, 204, 201, 153	Abscisic acid hexoside		5, 6, 9, 11
12	3.75	168	167	123, 108	Vanillic acid	C_8_H_8_O_4_	6, 9, 11
13	3.823	180	179	163, 135, 117	Caffeic acid	C_9_H_8_O_4_	5, 6, 9, 11
14	4.044	272	271	177,165,151,119,107	Naringenin	C_15_H_12_O_5_	5, 6, 9
15	4.50	302	301	257, 229, 185	Tannic acid	C_14_H_6_O_8_	5, 6, 9, 11
16	5.890	354	353	191, 179, 135	Chlorogenic acid	C_16_H_18_O_9_	5,6
17	6.006	358	357	151, 136, 128	Pine ester alcohol	C_20_H_22_O_6_	5, 6, 9, 11
18	6.689	492	491	329, 311,149,135	Caffeoyl dihydroxyphenyl lactyl tartaric acid		5, 6, 9
19	6.954	326	325	163,147, 187, 146, 119	Coumaryl hexose	C_15_H_18_O_8_	5, 6, 9, 11
20	7.523	302	301	179, 151	Quercetin	C_15_H_10_O_7_	5, 6, 9, 11

## Data Availability

The original contributions presented in this study are included in the article. Further inquiries can be directed to the corresponding author.
